# Revealing hidden complexities of genomic rearrangements generated with Cas9

**DOI:** 10.1038/s41598-017-12740-6

**Published:** 2017-10-09

**Authors:** Katharina Boroviak, Beiyuan Fu, Fengtang Yang, Brendan Doe, Allan Bradley

**Affiliations:** Wellcome Trust Sanger Institute, Wellcome Genome Campus, Hinxton Cambridge, CB10 1SA United Kingdom

## Abstract

Modelling human diseases caused by large genomic rearrangements has become more accessible since the utilization of CRISPR/Cas9 in mammalian systems. In a previous study, we showed that genomic rearrangements of up to one million base pairs can be generated by direct injection of CRISPR/Cas9 reagents into mouse zygotes. Although these rearrangements are ascertained by junction PCR, we describe here a variety of unanticipated structural changes often involving reintegration of the region demarcated by the gRNAs in the vicinity of the edited locus. We illustrate here some of this diversity detected by high-resolution fibre-FISH and conclude that extensive molecular analysis is required to fully understand the structure of engineered chromosomes generated by Cas9.

## Introduction

Many human genetic disorders show underlying causal deletions or duplications of large genomic regions^1–3^. Previously, generating mouse models for these chromosomal rearrangements required a multistep process termed chromosome engineering^4–6^. Using these approaches a variety of important mouse models for human disease such as for DiGeorge, Smith-Magenis syndrome, Autism and Down’s syndrome were generated^7–10^. However, generating these mouse models involves multiple genetic manipulation steps in embryonic stem (ES) cells, two gene targeting events followed by a Cre-mediated *loxP* recombination. Cells with the desired engineered chromosome(s) are then used to construct germ line chimaeras. The process is time consuming and challenging in part because ES cells can loose their pluripotency during their extended periods in culture.

CRISPR/Cas9 is an efficient genome editing tool in mammalian cells and zygotes^11,12^ which has been successfully utilized to generate a variety of disease models^13,14^. The utility of this technology to generate deletions, inversions and duplications has also been established^15–17^. We described previously the generation of 1.1 Mb deletions and inversions in mice^18^ while another group reported deletions and duplications up to 24.5 Mb in rats^19^. Although these rearrangements could apparently be efficiently generated, allelic heterogeneity was observed. For example, in experiments designed to generate a 121.7 kb deletion in rats, one founder was identified with a larger than intended deletion while the precise nature of the rearrangement in three animals could not be identified, even though they exhibited the expected phenotype^19^.

In our original study, we generated a series of rearrangements around the tyrosinase (*Tyr*) gene of up to 1.1Mb^18^. Overall, deletions and inversions were detected in 22–23% of founders, while duplications were identified infrequently, in just 2 out of 162 founders. We also observed mosaicism and loss of primer binding sites. For example, from 46 pups born for the *Nox4* rearrangement (155.3 kb region), six (13%) showed an inversion with only one junction PCR being positive and three (7%) showed mosaicism carrying a wild type or indel allele, a deletion as well as an inversion allele^18^.

To accurately interpret a phenotype it is critical to understand the molecular detail of the responsible allele(s). Genetic changes induced by the activity of Cas9 and a single guide RNA resolve into a spectrum of alleles while the combined activity of multiple guides increase the complexity further. In cases where the desired outcome is a structural rearrangement spanning one or more genes, short-range junction PCR assays of predicted junctions are helpful indicators of the fidelity of the genetic alteration, but the imprecise nature of these joins can lead to incorrect conclusions about the overall structure of the allele. Larger alleles are potentially more prone to this problem because of the difficulty of defining genome structure over long distances. In this report we demonstrate that in cases where large fragments are excised by pairs of guides these may reintegrate into the genome. The presence of a genetic scar generated by resolving ends left by an excised fragment, can give the impression that a “simple” deletion has been generated. Often however, the deleted fragment reintegrates close to the excision site where it can potentially restore the activity of some or all of the excised genes leading to an erroneous conclusion that the “deletion” has little or no phenotypic consequence.

## Results

The generation of mice with large chromosomal rearrangements using four gRNAs has been described previously^18^. Short-range junction PCR assays identified several mice which apparently carried a deletion or inversion, but in some cases just one of the junctions was completely verified while the other couldn’t be amplified and thus had an unresolved structure. Moreover, founders frequently exhibited mosaicism, with inversion and deletion alleles being detected in the same mouse.

**Figure 1:**
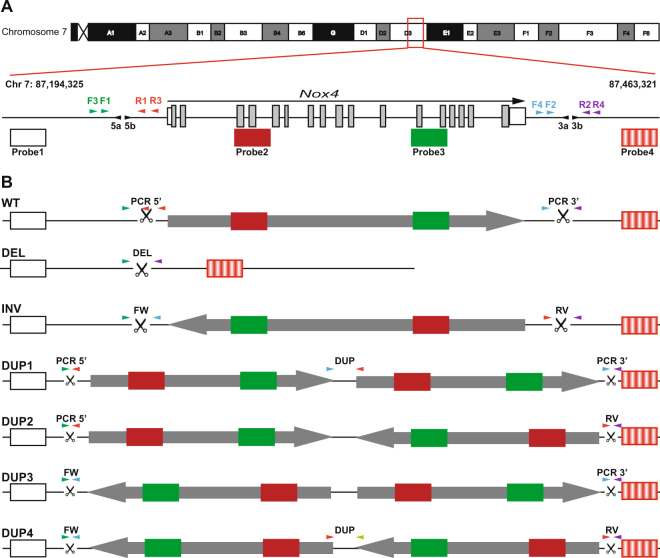
Overview of the 155.3 kb rearrangement region targeting *Nox4*. **(A)** The position of the gRNAs 5a, 5b, 3a and 3b are shown as black arrowheads. Four FISH probes across the *Nox4* region are illustrated, two are external to the gRNA cut side and two are within the *Nox4* gene. Primers for junction PCRs are depicted as arrowheads with the forward primers (F) marked green/blue and the reverse primers (R) being red/purple. **(B)** Schematic showing the different types of rearrangements that could be present within a founder animal, the corresponding PCRs used to identify them and the colour and order of the FISH probes.

To fully resolve the detailed spectrum of structural rearrangements generated in this previous study a representative cohort of founder and F1 mice derived from experiments designed to generate a 155.3 kb rearrangement (spanning *Nox4*) were examined, Fig. 1A﻿ and Table 1. These mice were subject to extensive short-range junction PCR assays to distinguish different rearrangements, Fig. 1B and Supplementary Fig. 1. Where an amplicon was generated this was sequenced. Additionally, fluorescent *in-situ* hybridization (FISH) analysis of metaphases, interphase nuclei and fibres was used to provide further information about the allele structure, Fig. 1 and Supplementary Fig. 1. Four fosmid probes were used, two external references positioned 12.3 kb and 23.7 kb from the gRNA target sites and two internal probes positioned between the gRNA sites. The interpretations of the FISH and junction PCRs for the expected alleles are shown, Fig. 1B.

### Mice with deletion junction fragments do not necessarily harbour deletions

**Figure 2:**
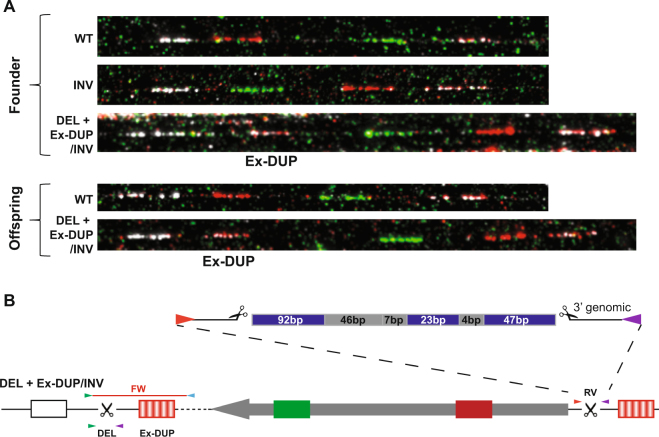
Characterization of founder mouse #1 and its offspring mouse #2. **(A)** Fibre-FISH show the presence of wild type (WT) and inversion (INV) alleles as well as an allele showing a deletion and the inversion of the deleted *Nox4* fragment as well as a duplication [Ex-DUP] of a region external to the gRNA cut site (DEL + Ex-DUP/INV). This allele was transmitted to offspring, mouse #2. **(B)** Representation of the DEL + Ex-DUP/INV allele. PCR primers F1/3 (green arrowhead) and F2/4 (blue arrowhead) are too far apart to generate a FW PCR product. The RV PCR has a 219 bp insertion between the 5′ and 3′ breakpoints consisting of fragments of sequences originating from either the bridge oligonucleotide (blue) or *Nox4* (dark grey). The junction between the external duplication and the inverted Nox4 region is shown as a dotted line.

Founder mouse #1 appeared to be mosaic as it was positive for both deletion and inversion junction PCRs although the inversion junction was identified at one end only (RV), Table 1. Surprisingly these alleles did not segregate in the founder’s progeny, rather both the deletion and inversion were co-inherited in 6 out of 8 pups born (75%) suggesting that they were linked on the same chromosome. Fibre-FISH analysis performed on the founder and an F_1_ pup revealed that the allele classified as a “deletion” also carried an inversion of the deleted segment and had duplicated probe 4 (Ex-DUP), a region external to the gRNA cut site, Fig. 2A and B. Additionally, the founder also carried an inversion allele which was not transmitted to its offspring, Fig. 2A.

**Table 1:** Overview of mice analyzed using endpoint PCR.

Junction	# 1	# 2 F_1_ of # 1	# 3	# 4	# 5	# 6	# 7	# 8	# 9 F_1_ of # 8
PCR 5′	faint	faint	+	+	+	+	+	double	+
PCR 3′	+	+	+	+	+	+	+	+	+
DEL	+	+	+	+					
FW			+						
RV	+	+		+					
DUP								+	+
PCR genotype	WT, DEL, INV RV	WT, DEL, INV RV	WT, DEL, INV FW	WT, DEL, INV RV	WT	WT	WT	WT, DUP1	WT, DUP1

+ indicates PCR positive.

Sequence analysis of the junction PCR product in both the founder and F_1_ showed that the deletion junction contained the bridging oligo although the sequence had a single nucleotide deletion, which we interpret as a synthesis error. The working junction PCR (RV, see Figs 1 and 2) which detected the inversion showed insertion of multiple short fragments that originate from *Nox4* as well as the bridging oligo used to facilitate the deletion Fig. 2B. The junction PCR for the other end of the inversion (FW) did not yield an amplicon. FISH analysis illustrates why it would not possible to amplify this fragment as the inverted DNA fragment had inserted telomeric to FISH probe 4. If the region corresponding to this duplicated probe is intact, the FW junction PCR would have to span at least 40 kb, Fig. 2B and Supplementary Fig. 1A.

**Figure 3:**
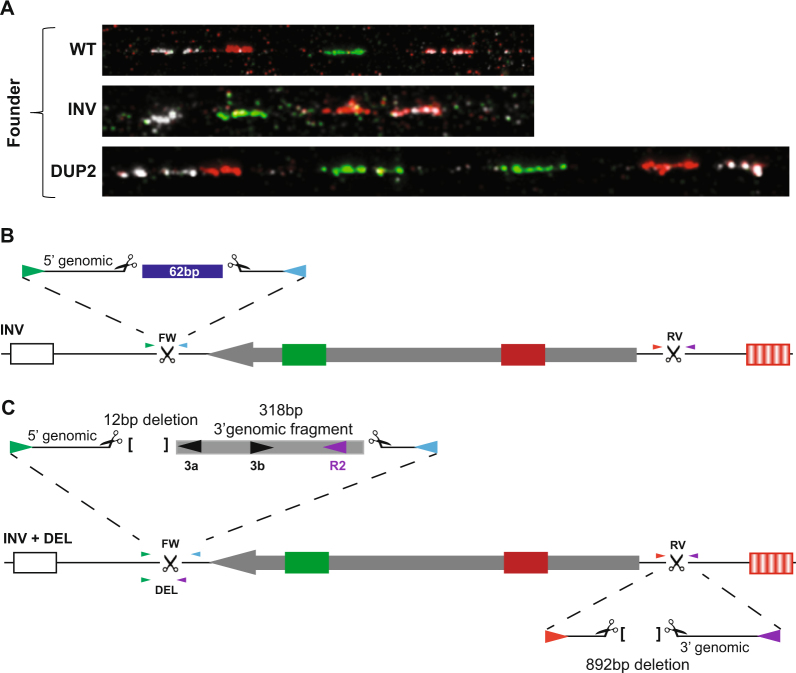
Analysis of founder mouse #3. **(A)** Fibre-FISH showing the presence of a wild type (WT), inversion (INV) and duplication (DUP2) allele in the founder. **(B)** Schematic of the INV allele detected in the founder as well as in one F1 offspring. The FW junction has a 62 bp insertion of the bridge oligo (blue). The RV junction for this allele did not yield a PCR product in the offspring. **(C)** Schematic of the second inversion allele with the deletion junction being present within the FW PCR fragment. Sequence of the FW PCR in the founder as well as three offspring revealed that the R2 primer (purple arrowhead) was present within the FW PCR fragment. Sequence of the RV PCR identified a 892 bp deletion removing both the R1 and R2 primer binding sites.

Founder #3 was also positive by PCR for a deletion and one end of an inversion (FW), Table 1. Fibre-FISH analysis on this animal revealed fibres with an inversion and an inverted duplication (DUP2) but no deletion was observed, Fig. 3A. Breeding of the founder resulted in the segregation of three different genotypes. Mice which were PCR positive for both an inversion (FW) and a deletion (3 out of 6 pups born), mice which were only positive for the inversion (FW) (1 out of 6 pups born) as well as wild type mice. Progeny with a duplicated inversion were not detected by junction PCR.

The single offspring that was PCR positive for the FW inversion amplicon had an insertion of 62 nucleotides originating from the bridging oligo at this breakpoint. However, the other inversion endpoint from this mouse could not be amplified which is presumed to be caused by loss of sequence corresponding to one or both PCR primer sites, Fig. 3B. Sequence analysis of the FW junction PCR from the three offspring that were positive for both deletion and inversion PCRs revealed that the R2 primer was present within this fragment, Fig. 3C. Thus, the deletion PCR product correctly diagnosed that a deletion had been generated on this chromosome, however the excised product had re-integrated immediately distal to the successfully repaired deletion breakpoint, albeit in an inverted orientation. The RV junction PCR only resulted in a product using the two external primer pairs (Fig. 3C and Supplementary Fig. 1). Sequence analysis of this RV junction PCR fragment revealed a 892 bp deletion which resulted in the loss of primers R1 and R2, Fig. 3C.

### Distinguishing inversions from duplications

**Figure 4:**
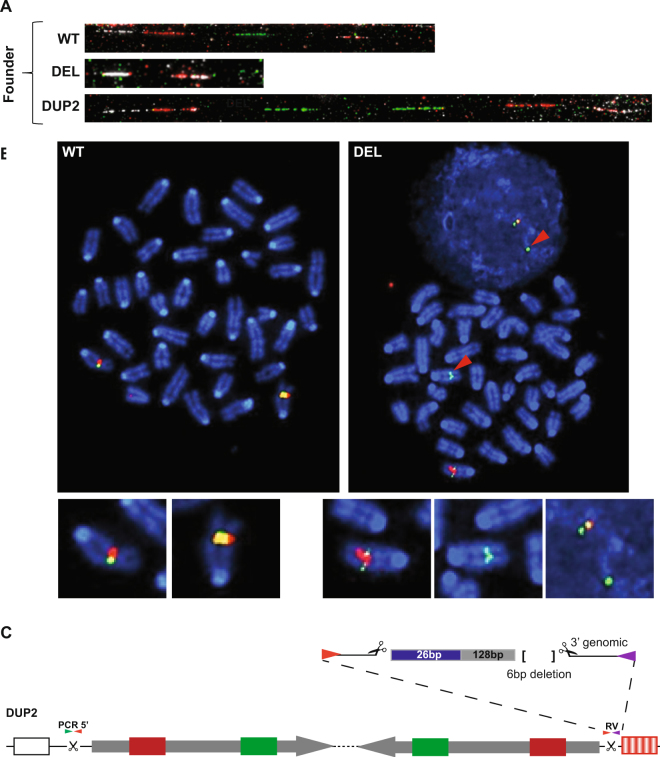
Analysis of founder mouse #4. **(A)** Fibre-FISH showing presence of a wild type (WT), deletion (DEL) and duplication (DUP2) allele. **(B)** Meta and interphase-FISH with two colours; probes 1 + 4 (external) are green while probes 2 and 3 (internal) are red, confirming the DEL allele (red arrowheads). **(C)** Illustration of the structure of the duplication allele (DUP2) observed in this mouse. Sequence of the RV PCR band identified a 26 bp insertion from the bridge oligo (blue), 128 bp of *Nox4* (grey) and a 6 bp deletion. The junction between the inverted duplicates is shown as a dotted line.

Founder mouse #4, had a PCR genotype which suggested it carried a deletion and an inversion, indistinguishable by PCR from founder #1 (Table 1). Sequence analysis confirmed that the deletion contained the bridging oligonucleotide. Analysis of the working inversion junction PCR (RV) revealed that it was resolved from two break-repair events with a 26 bp insertion (originating from the 5′ arm of the oligonucleotide), 128 bp of *Nox4* and a 6 nucleotide deletion, Fig. 4C.

FISH confirmed that the majority of alleles present (81 of 100 analysed, 81%) in this founder were deletion alleles, Fig. 4B. However, fibre-FISH also showed the presence of an inverted duplication instead of the expected inversion, Fig. 4A. The duplicated inverted fragment is inserted telomeric to what appears to be the original copy of the locus, an arrangement which we refer to as DUP2, Fig. 1B. Although the inversion was detected by PCR, the duplication junction between the *Nox4* duplicates was not, presumably because one or both primer binding sites had been lost and/or a large fragment of exogenous DNA was inserted between them. The most centromeric junction (from the non-inverted duplicate, PCR 5′) may either be unchanged so it would amplify as a wild type allele, or it may also have been altered beyond the possibility of PCR detection.

### FISH improves detection and resolution of structural changes

Given the imperfect correlation between the PCR and FISH genotypes we also analysed three mice that were genotyped as wildtype by short-range junction PCR, Supplementary Fig. 2. Just one was confirmed to be wild type, Supplementary Fig. 2A. The remaining two mice carried a deletion (Supplementary Fig. 2B) and a duplication (Supplementary Fig. 2C) respectively, with mouse #7 possibly showing a partial duplication of the region illuminated by the red probe. The failure to detect these alleles by PCR, likely reflects loss of the PCR primer sites and/or insertion of sequence between them making the products too long for short-range junction PCR.

Fibre-FISH also revealed that some of the rearrangements were not intact. For example, one founder and its offspring genotyped as carrying a duplication by PCR were discovered to only carry a partial duplication by fibre-FISH as the signal was detected with just one of the two internal probes, Supplementary Fig. 3. Using only short-range junction PCR this partial duplication would have only been noticed in a homozygous line as the 3′ PCR detects the wildtype allele which is present in both the founder and heterozygous animals and thus would have only been lost in homozygous animals. We also analysed some mice from the 1 Mb rearrangement (spanning *Nox4*, *Tyr* and *Grm5*)^18^ as well as a 664 kb rearrangement on mouse chromosome 1 (spanning from *Cfhr1 to Cfh*) and found similar events (data not shown).

## Discussion

In this study we examined founders and descendants of zygotes injected with two pairs of gRNAs spanning the *Nox4* gene, a distance of 155.3 kb. Short-range junction PCR analysis was used to identify mice that were believed to carry deletions, inversions and duplications. In many cases the results were ambiguous, particularly in the founders which often carried more than one rearrangement. While in a number of cases segregation of the alleles in descendants enabled the identification of mice with the desired structural rearrangement, the structure of the rearrangement in many cases was unclear because only one of the breakpoints was recovered. Fibre-FISH analysis was used to try and resolve some of this ambiguity. This not only revealed an unprecedented level of complexity but also demonstrated the limitations of the extrapolation of conclusions from short-range junction PCR assays to a larger genomic region.

Short-range junction PCR was believed to provide compelling evidence that a desired genomic alteration has been generated; for instance in the case of a deletion the juxta-position of sequences that were previously 155.3 kb apart. The extrapolation of junctional information to the inference that the intervening sequences are intact is founded on the analysis of rearrangements generated with Cre/loxP^20^. The evidence presented here demonstrates that such assumptions are not valid when Cas9 is used to generate similar changes.

Cas9 has numerous mechanistic differences from Cre, emanating from the evolutionary pressures which have driven the development of these enzymes. Cre has evolved to recombine two distant *lox* sites in the P1 phage genome while keeping the intervening sequences intact, achieved by forming a DNA-protein complex with both recognition sites and four molecules of Cre before the reaction proceeds. The exchange reaction between lox sites thus proceeds in the context of intimate adjacency of intended targets of the recombination event and is precise to the nucleotide, enabled in part by covalent attachment of Cre to the cut DNA in the process of mediating the molecular exchange^20^. By contrast, Cas9 has evolved to destroy invading DNA and thus aside from sequence recognition of its DNA target it but does not participate in any repair process^21–23^. When Cas9 is used to affect a structural change one is entirely reliant on the host DNA repair machinery to re-join broken DNA ends. In the context of chromosome engineering we make the assumption that the two broken DNA fragments are not only generated in the same temporal context, but that they are able to find each other to be re-joined by the host repair machinery. Moreover, we assume that the excised fragment of DNA is degraded or otherwise lost.

A single genomic site cut by Cas9 is re-joined by the imprecise process of non-homologous end-joining (NHEJ)^11,12,24,25^. In such a circumstance, re-joining is facilitated by the adjacency of the DNA ends. In cases where two chromosomal breaks are generated some distance apart there are a variety of potential outcomes, ranging from simple NHEJ of each of the two ends to structural alterations that encompass the DNA fragment defined by the gRNA target sites. The zygote is diploid and is actively replicating its DNA at the time of injection, thus there may be as many as four copies of the target present. Given this, it is perhaps not unexpected that significant allelic complexity arises from a conceptually simple editing experiment.

Prior to this study we assumed that if a deletion junction was detected by junction PCR, the excised DNA between the gRNA target sites was lost from the genome^18^. Indeed, in our previous study we identified founders and F_1_’s by short-range junction PCR and FISH that carried the designed deletion. Inversions and duplications were also identified, however, many founders were mosaic and/or only one junction could be confirmed by PCR^18^. The results presented here illustrate that the excised fragment can re-integrate locally. In such a circumstance the PCR may detect the correct deletion junction, but the re-inserted fragment can potentially restore the activity of some or all of the excised genes leading to an erroneous conclusion that the “deletion” has little or no phenotypic consequence. In another example, junction PCR suggested a duplication had been formed, however fibre-FISH revealed that only part of the locus had been duplicated. In yet other cases, even though the mice were genotyped as wildtype by junction PCR, fibre-FISH revealed they carried a variety of structural changes. We also identified one founder and its offspring in which DNA external to the region demarcated by the gRNA had been duplicated.

The molecular details of an allele must be fully defined to interpret a phenotype. Although a spectrum of alleles is generated by NHEJ following Cas9 cleavage of a single genomic site, the resultant genomic scars are relatively small in size and comparatively simple to characterize at the nucleotide level. In contrast, when multiple gRNAs are used to construct structural re-arrangements, the alleles generated are significantly more complex. Although short-range junction PCRs are helpful to classify the type of rearrangement they do not yield a complete understanding of the structural change. The characterisation of large-scale structural changes generated by an unpredictable process is formidable, particularly where the region involved is very large. Moreover, certain types of rearrangement present significant challenges, such as duplications of the fragment itself and sequences external to the fragment. Re-integration of the excised DNA outside the immediate area of focus can not only restore gene function of all or part of the locus, but the insertion itself can disrupt flanking genes. In this study we show that fibre-FISH is able to illuminate some of this complexity. However, the resolution of this technique is limited and precise details such as the definition of all junctions, breakpoints and ideally the sequence of the entire locus are required to fully understand the alleles generated by this technique. Moreover, extreme care should be taken when litter mates that are believed to be wildtype are used as controls.

As a chromosome engineering tool, Cas9 offers the apparent advantage of speed compared with the previous technology Cre-*loxP*. However, the effort and difficulty in unambiguously defining the structure of the alleles which emerge from an experiment over considerable genomic distances is considerable. In contrast, although chromosome engineering conducted with Cre-*loxP* is a multistep process conducted in ES cells, the resulting structure is totally predictable. Thus, although Cas9 can rapidly generate genomic rearrangements of significant size, understanding their structure may ultimately involve more time and effort than using Cre-*loxP*.

## Materials and Methods

### *In vitro* transcription of gRNAs and Cas9 mRNA

CRISPR/Cas9 target sites were identified using http://crispr.mit.edu/ as well as http://bioinfogp.cnb.csic.es/tools/breakingcas/. In addition the guide RNAs were picked following the guidelines from Doench, *et al*.^26^ avoiding C and T upstream of the PAM, G downstream of the PAM and T within the PAM whenever possible. Pairs of gRNAs were designed for each endpoint which were typically located within 50 to 200 bp of each other and positioned on opposite strands (PAM out orientation) whenever possible (Supplementary Table 1). For Cas9 mRNA production, the T7/Cas9 plasmid^18^ was linearized with EcoRI and for gRNA production with DraI. The plasmids were cleaned with a PCR purification kit (Quiagen) and *in vitro* transcribed using mMessage mMachine T7 Ultra kit and MEGAshortscript T7 kits (Life Technologies), respectively. Both, Cas9 mRNA and gRNA, were purified using the MEGAclear kit (Life Technologies) and eluted in RNase-free water. The quality of the RNA was analysed using Agilent RNA 6000 Nano kit (Agilent Technologies, 2100 Bioanalyzer) and Qubit RNA HS assay kit (Life Technologies).

Single strand oligonucleotides (ssODN) designed to bridge the deletions were 120 bp in length, positioned directly adjacent to the most external gRNA site and contained a NotI restriction site (Supplementary Table 1). The single strand oligonucleotides (ssODN) were synthesized by Integrated DNA Technologies (IDT) and dissolved in RNAse-free water to a concentration of 1000 ng/ul.

### Zygote injection

4-5 week old C57BL/6NTac females were super-ovulated by intraperitoneal (IP) injection of 5 IU of pregnant mare’s serum (PMSG) at 12:00–13.00 hrs (on a 12 hr light/dark cycle, on at 07:00/off at 19:00) followed 48hrs later by an IP injection of 5 IU human chorionic gonadotrophin (hCG) and mated overnight with C57BL/6NTac stud males. The next morning the females were checked for the presence of a vaginal copulation plug as evidence of successful mating, oviducts were dissected at approximately 21–22 hrs post HCG and cumulus masses retrieved and treated with hyaluronidase as previously described^27^. Fertilized 1-cell embryos were selected and maintained at 37 °C in KSOM media prior to cytoplasmic injection. Injections were carried out between 24–27 hrs post HCG.

50 ng/ul Cas9 mRNA, 25 ng/ul gRNA (total) and 100 ng/ul oligonucleotide were mixed in RNase free water, backfilled into an injection needle with positive balancing pressure and injected into the cytoplasm of fertilized 1-cell embryos held in FHM medium. Injected embryos were briefly cultured to check for embryo viability post injection and surviving embryos were transferred the same day by oviducal embryo transfer into a 0.5 days post coital pseudo-pregnant female F1 (CBA/C57BL/6 J) recipients^27^.

All procedures performed in studies involving animals were in accordance with the ethical standards of the institution or practice at which the studies were conducted and performed with approval of the UK home office.

### DNA isolation and genotyping of mutant founders and their offspring

Genomic DNA was isolated from ear clips of F_0_ founder mice and their offspring, using the Sample-to-SNP kit lysis buffer (Life Technologies). For short-range junction PCR, 1ul of the ear clip lysate was used per PCR reaction with High Fidelity Platinum Taq polymerase (Life Technologies). The PCR products were examined on gels and sequenced to ascertain the integrity of inversions, duplications and deletions (Supplementary Table 2).

### Fluorescent insitu hybridization

Fosmid clones used for FISH were provided by the clone archive resource of Wellcome Trust Sanger Institute. Metaphase-FISH essentially followed Gribble *et al*.^28^. Fiber-FISH was performed as described previously by Perry *et al*.^29^ with some modifications. Briefly, extended chromatin and DNA fibres were prepared by alkaline lysis from mouse splenocytes. Fosmid DNA was labelled using biotin-16-dUTP, digoxigenin-11-dUTP, dinitrophenol (DNP)-11-dUTP (Jena Bioscience) following the protocol detailed in Louzada *et al*.^30^. Biotin labelled probe was detected with Cy3-streptavidin (Sigma-Aldrich); Digoxigenin-labelled probe was detected with monoclonal mouse anti-DIG IgG (Sigma-Aldrich) and Texas red conjugated donkey anti-mouse IgG (Invitrogen); DNP-labelled probe was detected with rabbit anti-DNP and Alexa 488 conjugated goat anti-rabbit IgG. After detection, slides were mounted with SlowFade Diamond® (Invitrogen) mounting solution containing 4′,6-diamidino-2-phenylindole (Invitrogen). Images were captured on a Zeiss AxioImager D1 fluorescent microscope and processed with the SmartCapture software (Digital Scientific UK). The probes used in this study are summarized in Supplementary Table 3.

The datasets generated during and analysed during the current study are available from the corresponding author on reasonable request.

## Electronic supplementary material

Supplementary Information
