# Author Correction: Revealing hidden complexities of genomic rearrangements generated with Cas9

**DOI:** 10.1038/s41598-018-23232-6

**Published:** 2018-04-05

**Authors:** Katharina Boroviak, Beiyuan Fu, Fengtang Yang, Brendan Doe, Allan Bradley

**Affiliations:** Wellcome Trust Sanger Institute, Wellcome Genome Campus, Hinxton, Cambridge, CB10 1SA United Kingdom

Correction to: *Scientific Reports* 10.1038/s41598-017-12740-6, published online 09 October 2017

The original version of this Article contained an error in the Abstract.

“Although these rearrangements are ascertained by junction PCR, we describe here a variety of anticipated structural changes often involving reintegration of the region demarcated by the gRNAs in the vicinity of the edited locus.”

should read:

“Although these rearrangements are ascertained by junction PCR, we describe here a variety of unanticipated structural changes often involving reintegration of the region demarcated by the gRNAs in the vicinity of the edited locus.”

This error has now been corrected in the PDF and HTML versions of the Article.

