# Supplementary material for: Revealing hidden complexities of genomic rearrangements generated with Cas9
Source: Sci Rep. 2017 Oct 9;7:12867. doi: 10.1038/s41598-017-12740-6 (PMC5634419; doi:10.1038/s41598-017-12740-6)
Supplement: Supplementary file 1 — Supplementary Information [file 41598_2017_12740_MOESM1_ESM.pdf]

Revealing hidden complexities of genomic rearrangements generated with Cas9

Katharina Boroviak, Beiyuan Fu, Fengtang Yang, Brendan Doe & Allan Bradley

Wellcome Trust Sanger Institute,

Wellcome Genome Campus,

Hinxton, Cambridge United Kingdom

CB10 1SA

Supplementary Information

Supplementary Figures

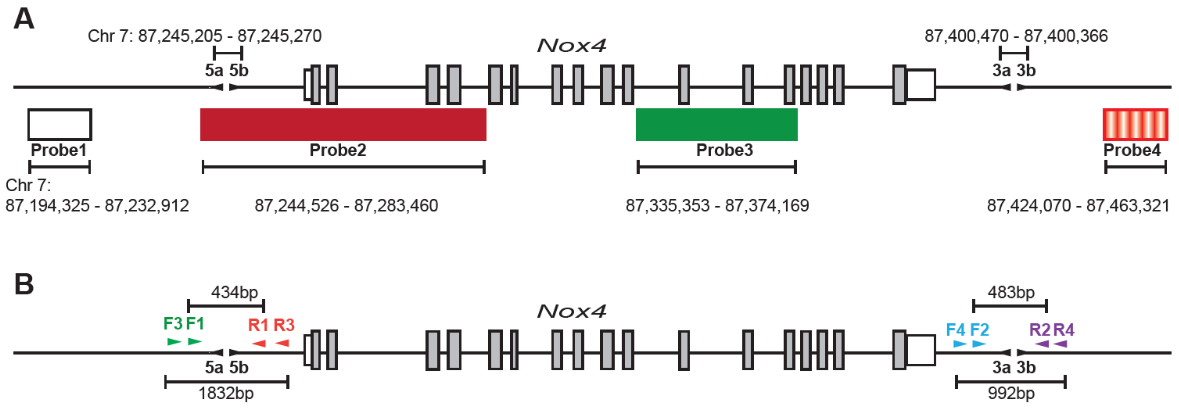

Supplementary Figure 1. Schematic of fosmid probes and PCR primers. (A) Chromosomal locations for gRNAs and FISH fosmid probes. **(B)** Expected wild type PCR product sizes resulting from the most internal (top) and external (bottom) pairs of PCR primers.

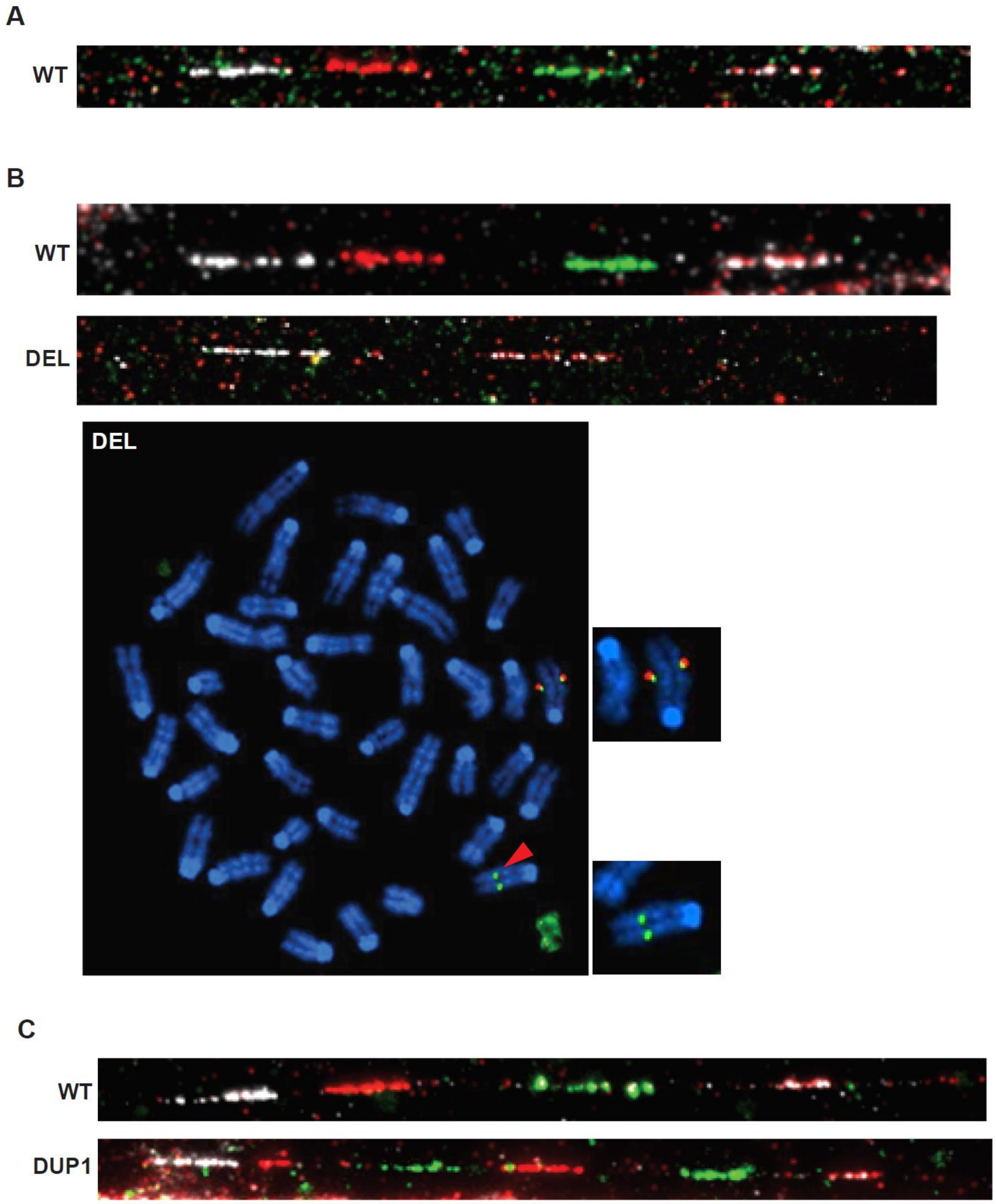

Supplementary Figure 2. Analysis of founder mice that had a wild type genotype by breakpoint PCR. (A) Mouse #5 was confirmed to be WT. **(B)** Mouse #6 showed unmodified (WT) and deletion (DEL) alleles by fibre-FISH which was confirmed using metaphase/interphase-FISH. **(C)** Mouse #7 had unmodified (WT) and duplication (DUP1) alleles by fibre-FISH with the first red probe appearing to be slightly shorter, indicating a possible partial duplication.

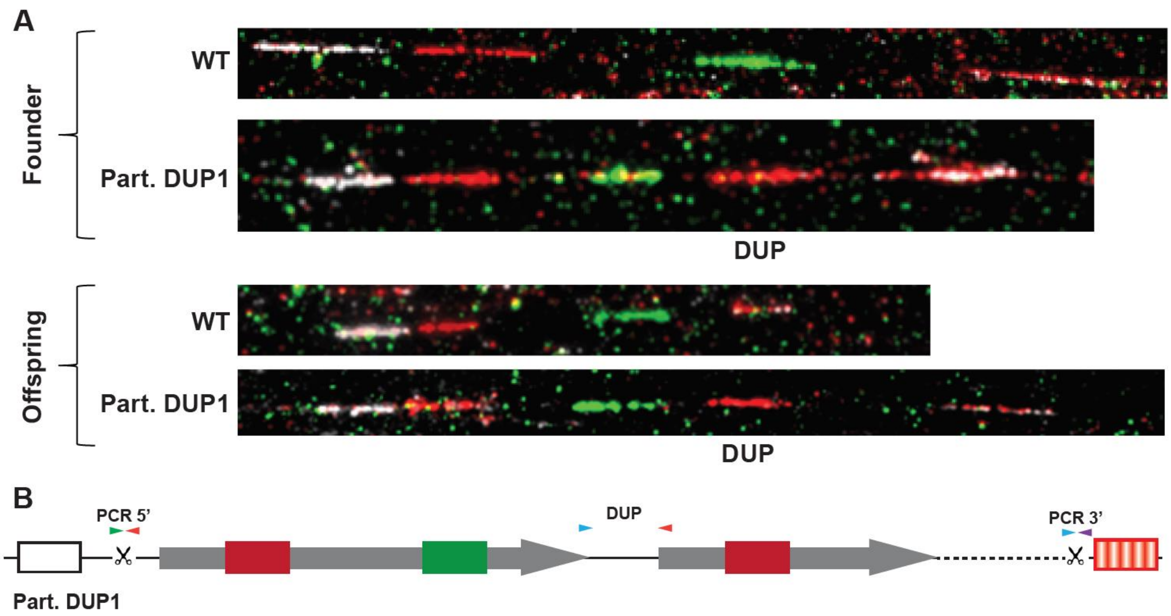

Supplementary Figure 3. Analysis of mouse founder #8 and its offspring, mouse #9. (A) Fibre-FISH analysis of founder #8 showing presence of a partial duplication allele (Part.DUP1) which was transmitted to its progeny mouse, below. **(B)** Illustration of the structure of the partial deletion allele with the second probe 3 (green probe) missing. The junction between the duplication and the 3'end is shown as dotted line.

Supplementary Tables

Supplementary Table 1: Guide RNA and oligo sequences for Nox4 rearrangement

deletion	gRNA	sequence (5' - 3')	coordinates	bridging oligo sequence (5' - 3')
155kb	5a	AAAACTCCAAGTTGGGATGCAGG	7:87,245,205-87,245,227	CAACCTGGTCTTCATGTGGGTTCCAAAAATTTGGAGTGAAGGCTATCCCAAAAGCTGTTGG CGGCCGCAGATTTGCATCTGAGAAGTTATCTGCTGAACTTGCTCCTCCTATCCTGGA
	5b	CAGCCTCTCATTTGGGGCATGGG	7:87,245,248-87,245,270	
	3a	GCTCCGAGGAAAAGTCGAGGGGG	7:87,400,470-87,400,492	
	3b	GACATATCCCTTATATATACAGG	7:87,400,344-87,400,366	

All sequences are from 5' to 3'; PAMs are undelined.

Supplementary Table 2: Genotyping primer sequences for endpoint PCR

primer	sequence (5' - 3')	coordinates
F1	AACTCAAAATAGTTTCCTGGACATC	7:87,245,081-87,245,105
F3	TTCAGACATGGGCAGACAATCA	7:87,244,419-87,244,440
R1	TCTCAAGCATGAGTGAGTCAAGA	7:87,245,492-87,245,514
R3	TCCAGCCAGTCCTGTGTCTA	7:87,246,231-87,246,250
F2	AAAGGACCCTGCTCAAGACTC	7:87,400,142-87,400,162
F4	GCATGTATACAGAGAGAGAGTGAG	7:87,399,873-87,399,896
R2	AGAGTCTGAACACCCAACCA	7:87,400,605-87,400,624
R4	GCAAGCACCAATCCCTGACA	7: 87,400,844-87,400,864

Supplementary Table 3: location of FISH probes

Probe	Label	Whitehead Institute ID	Clone Name (UCSC)	Start	End
1	Biotin	WI1-0437J6	G135P67344D10	87,194,325	87,232,912
2	Dig	WI1-0344C7	G135P63059G9	87,244,526	87,283,460
3	DNP	WI1-0630H13	G135P6566E6	87,335,353	87,374,169
4	Biotin & Dig	WI1-1026A5	G135P600291H10	87,424,070	87,463,321
